# Studies on the effect of adenosine on calcium oscillation in hippocampal neurons

**DOI:** 10.3892/etm.2013.973

**Published:** 2013-02-25

**Authors:** JINBO CHEN, YUAN WANG, YULIANG WANG, XIANGMING YI, RULI GE

**Affiliations:** Department of Neurology, The Affiliated Hospital of Binzhou Medical University, Binzhou 256603, P.R. China

**Keywords:** adenosine, confocal laser scanning microscope, calcium oscillation

## Abstract

Adenosine (Ade) is an antiepileptic agent. In order to investigate the possible mechanism of action of Ade, its effect on calcium (Ca^2+^) oscillations in hippocampal neurons of Sprague Dawley (SD) rats was explored. Primary hippocampal neurons were cultured from suckling neonatal SD rats. Cells were cultured for 7–9 days and the Ca^2+^ oscillations in response to perfusion with Ade were detected using confocal laser scanning microscopy in combination with Fluo-3/AM labeling. This study found that Ade inhibits the spontaneous synchronized Ca^2+^ oscillation frequency and amplitude in mature hippocampal neurons and such inhibition depends on the Ade dosage level to a certain extent. Ade also had a significant inhibitory effect on high potassium-induced Ca^2+^ oscillation frequency and amplitude. Ade had a significant inhibitory effect on high-voltage-activated Ca^2+^ channel-mediated Ca^2+^ influx and Ca^2+^ oscillations in neurons. This may be one of the mechanisms for Ade to exert antiepileptic effects as an endogenous substance.

## Introduction

Calcium (Ca^2+^) oscillation refers to the temporal and spatial undulation of Ca^2+^ concentration and is indicative of synchronized electrical activities in the neuronal network. The electrophysiological characteristics of an epileptic attack mainly include hypersynchronous discharge of neurons of the local or whole brain; therefore Ca^2+^ oscillations have always been considered as a simple mode of electrophysiological movement of the epileptic nerves. At present, the biggest problem in epilepsy treatment is the insensitivity to existing exogenous substances (1). The antiepileptic nature of adenosine (Ade) has attracted much attention. Ade is the intermediate product of energy metabolism widely existing *in vivo*, so if endogenous Ade can be successfully induced to exert its antiepileptic effect, an effective and new treatment method will be provided for drug-resistant patients with refractory epilepsy. In this sense, a study of the relationship between Ade and Ca^2+^ oscillations has important clinical significance. This study aims to observe the effect of Ade on Ca^2+^ oscillations in the nerve cells of primary cultured hippocampal neurons *in vitro* with confocal laser scanning microscopy. The hippocampal tissue is implicated in epilepsy and if Ade affects the electrical activities between the neural networks it may explain the antiepileptic mechanism of Ade.

## Materials and methods

### Animals

Neonatal Sprague Dawley (SD) rats (no more than 48 h old) provided by the Experimental Animal Center of Binzhou Medical University were randomly selected without restriction of gender. The study was carried out in strict accordance with the recommendations in the Guide for the Care and Use of Laboratory Animals of the National Institutes of Health. The animal use protocol was reviewed and approved by the Institutional Animal Care and Use Committee (IACUC) of the Affiliated Hospital of Binzhou Medical University.

### Culture of primary hippocampal neurons

The primary hippocampal neurons were cultured referring to related studies (2,3). The specific steps were as follows. Neonatal SD rats were disinfected with 75% alcohol. Their cerebral hemispheres were dissected out, the olfactory bulbs retracted from the front with an ophthalmological forceps perpendicular to their brainstems, and the cerebra removed. Their cerebral cortices were opened on both sides along their longitudinal cerebral fissures to show the semilunar hippocampus. Each separate hippocampus could be seen on both sides. The membrane, blood vessel and non-hippocampal formation were carefully removed and the hippocampus was washed with Hank’s Balanced Salt Solution (HBSS). The hippocampus was cut into 3×1 mm pieces and added to ∼2 ml of 0.125% trypsin (Gibco-BRL, Carlsbad, CA, USA). The mixture was gently shaken and digested for 10–15 min in an incubator at 37°C. On completion of digestion, the pancreatin was removed using a Pasteur pipet. The digestion was then terminated for 5 min with 5 ml of the planting medium. The tissue suspension was gently blown with a polished pipet ∼20 times and filtered with a 200 mesh screen. The filtration was transferred into the centrifuge tubes, and then centrifuged at 800 rpm for 4 min to remove the supernatant. The planting medium was added, then the mixture was made into cell suspension through blowing, and the cell suspension thus obtained was planted on the culture dish with a coverslip coated with polylysine (Gibco-BRL). The planting medium was added to each culture dish, so that the volume on each culture dish reached 2 ml, then the culture dishes were cultured in an incubator with 5% CO2 at 37°C. All media were replaced with maintenance media 24 h later. Afterwards, half of the liquid volume in the maintenance media was replaced once every three days.

### Experimental group

In order to record Ca^2+^ oscillations, the hippocampal neurons cultured with Krebs-Ringer solution as a basal medium were divided into 4 groups: control group, high potassium treament group, Ade treament group, high potassium and Ade co-treatment group. High potassium group: neurons were treated with 60 mmol/l KCl. Ade treament group: neurons were treated with Ade at 0.1 *μ*mol/l and 50 *μ*mol/l, seperately. High potassium and Ade co-treatment group: neurons were treated with 50 *μ*mol/l Ade and 60 mmol/l KCl. Control group: neurons were just treated with Krebs-Ringer solution.

### Ca^2+^ oscillation records by laser scanning confocal microscopy

The fluorescence intensity produced by a fluorescent probe Fluo-3/AM (Biotium Company, San Francisco, CA, USA) is proportional to the intracellular free Ca^2+^ concentration. Therefore, the hippocampal neurons were carried in Krebs-Ringer solution (final concentration of 6 *μ*mol/l) of Fluo-3/AM in an incubator with 5% CO_2_ at 37°C for ∼30 min, followed by quick flushing three times and then suspending in the recording solution for delipidation with Fluo-3/AM for ∼15 min (4). When the above steps were completed, the cells growing on 30-mm special slides were directly placed in the matching stainless steel tank, followed by image acquisition with an inverted fluorescence microscope (Olympus FV500, Japan). A 485-nm wavelength was selected to activate Fluo-3/AM. Here, a pre-cooled camera was used to acquire the necessary images, which were acquired and analyzed using the Fluoview Tiempo time course software. After adjusting to the desired concentration, Ade was administered with the perfusion device. Ca^2+^ oscillations in hippocampal neurons were recorded as follows: before addition of Ade, the oscillation was shot for ∼3 min; after addition of Ade, the oscillation was continuously shot for ∼12 min.

### Quantitative analysis method of Ca^2+^ oscillations (2)

Data were obtained from fluorescence images by analyzing the average fluorescence intensity in the pixel region of ∼3×3 in the center of the hippocampal neuronal cell bodies which had pyramidal shapes, well developed branches and good adherence. ΔF/F_0_, the relative fluorescence intensity change of Fluo-3 shows the intracellular Ca^2+^ concentration change, and is used to reflect the amplitude of Ca^2+^ oscillation. ΔF is the Ca^2+^ concentration at the moment of t and F_0_ is the average baseline value obtained within the unit time of t±10 sec. Ca^2+^ oscillation is defined when ΔF/F_0_ is dramatically increased by more than 20%. Ca^2+^ oscillation frequency and amplitude are obtained from calculating the frequency and average amplitude within the fixed unit time of 2 min, and are used as the statistical record of the experimental data.

### Statistical analysis

Data were analyzed using SPSS 11.0 (SPSS, Inc., Chicago, IL, USA) statistical software. Measured data were expressed as the means ± SEM using the paired t-test. P<0.05 was considered to indicate a statistically significant result.

## Results

### Identification of the hippocampal neurons

**Figure 1:**
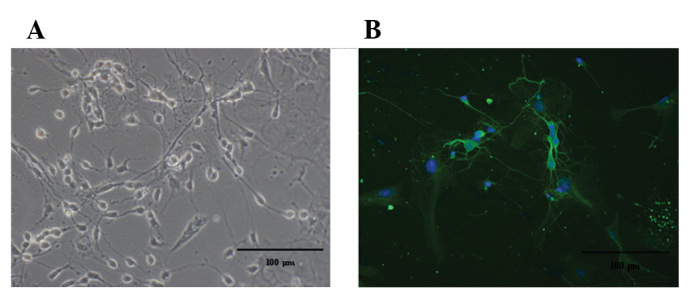
(A) Nine-day-old hippocampal neurons under confocal microscope (magnification ×400, scale 100 *μ*m). (B) Identification of hippocampal neurons (green) using immunofluorescence staining with Tubulin (scale 100 *μ*m).

Hippocampal neurons were observed with a high power microscope after 7–9 days using indirect immunofluorescence staining with Tubulin. Green represents neuronal matter and blue represents the nerve cell nucleus (Fig. 1).

### Ca^2+^ oscillation records

**Figure 2:**
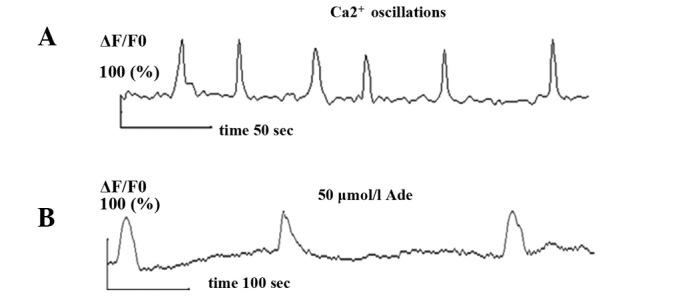
Randomly selected hippocampal neurons from a group of synchronized calcium (Ca^2+^) oscillations for recording. (A) Spontaneous synchronized Ca^2+^ oscillations in hippocampal neurons. (B) Effect of 50 *μ*mol/l adenosine (Ade) on spontaneous synchronized Ca^2+^ oscillations in hippocampal neurons.

Spontaneous synchronized Ca^2+^ oscillations were observed in primary cultured hippocampal neurons using confocal laser technology. Ca^2+^ oscillation frequency and amplitude were 1.2±0.12/min (0.02±0.002 Hz) and 1.87±0.17, respectively (Fig. 2A), which was consistent with literature (4).

### Effect of Ade on Ca^2+^ oscillations

Once dissolved in Krebs-Ringer solution to achieve the desired concentration, Ade could be tested for its effect on mature hippocampal neurons. Krebs-Ringer solution (1 ml) was prepared in a dish and observed for ∼3 min, which was taken as the control Ade intervention. The solution was then replaced by 1 ml freshly prepared Ade solution for continual recording and neurons maintained in 1 ml of Krebs-Ringer solution were used as a control. Following the addition of Ade, spontaneous Ca^2+^ oscillation frequency was measured at various times. The results showed that 0.1 *μ*mol/l Ade had no significant effect on Ca^2+^ oscillations but 50 *μ*mol/l Ade inhibited the spontaneous synchronized Ca^2+^ oscillation frequency and amplitude (n=12, P<0.05). Its frequency was reduced from 1.2±0.2/min (0.02±0.003 Hz) before addition of Ade to 0.3±0.05/min (0.005±0.001 Hz) and its amplitude was decreased from 1.87±0.17 before addition of Ade to 1.1±0.07 (Fig. 2B).

**Figure 3:**
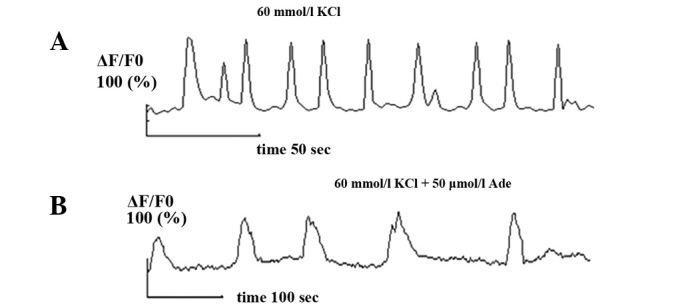
(A) Calcium (Ca^2+^) oscillation changes in hippocampal neurons after addition of 60 mmol/l high potassium solution. (B) Further changes in Ca^2+^ oscillations were observed with 50 *μ*mol/l adenosine (Ade) in the presence of high potassium solution for ∼3 min.

After perfusion of high potassium recording solution, the observed confocal results showed that Ca^2+^ oscillations were significantly enhanced in respect of frequency and amplitude. After recording for 3 min, the Ade (50 *μ*mol/l) intervention began and Ca^2+^ oscillation frequency and amplitude were inhibited again (n=7, P<0.05). The frequency was reduced from 2.39±0.22/min (0.04±0.003 Hz) before addition of Ade to 0.44±0.13/min (0.01±0.002 Hz) and its amplitude was decreased from 2.45± 0.27 before addition of Ade to 1.12±0.08 (Fig. 3).

## Discussion

Ade is the precursor and product of adenine nucleotide metabolism. Energy is used throughout the human body and this endogenous purine nucleoside is widely distributed in tissues all over the body. It is involved in the regulation of a variety of physiological functions by activating Ade receptors (A1, A2a, A2b, and A3) (5,6). In the central nervous system, Ade is a normal component of the extracellular fluid of neurons and has a low physiological level (0.03–0.3 *μ*mol/l). During an epileptic attack, however, the Ade concentration is increased by 6–31 times. Some researchers have reported that Ade secretion and Ade receptor expression were significantly decreased in the refractory epilepsy model (7). Some studies have also shown that transplantation of adenosine kinase (ADK)-knockout glial cells which secrete Ade was able to terminate an epileptic attack in animal models, suggesting that endogenous Ade exerts a natural antiepileptic effect. Knowledge of the target area and mechanism of action of Ade has important significance for further research on how to activate Ade to fully exert its antiepileptic effect. Jackisch *et al* found that the hippocampal neurons in the central nervous system are particularly sensitive to the effect of Ade (4), so a preliminarily discussion of the antiepileptic mechanism of Ade with primary cultured hippocampal neurons as the model is appropriate.

Ca^2+^ oscillation is the temporal and spatial undulation of Ca^2+^. The formation of Ca^2+^ oscillations within the neuron network is modified through extracellular Ca^2+^ influx in combination with the release and assimilation of intracellular Ca^2+^ stores. Ca^2+^ oscillation is an ubiquitous phenomenon in the nervous system tissues and Ca^2+^ can rapidly diffuse in several ways, including gap junctions. Its frequency and amplitude changes code neural network information and play an important role in synaptic plasticity and neuronal transfer (11). Koizumi and Inoue (12) reported that under physiological conditions, synchronous primary Ca^2+^ oscillations exist in the hippocampal neurons of rats; such Ca^2+^ oscillation is closely related to the synapse stimuli and can trigger excitation. Some experiments proved that the synchronization of neuronal electrical activity marked by the Ca^2+^ oscillation is crucial to the propagation of epileptiform discharges (13). The release of epileptiform activity starts with the intrinsic burst discharge in neurons, wherein the excessive influx of Ca^2+^ passing through the Ca^2+^ channels and intracellular Ca^2+^ store release plays an important role. At the same time, Ca^2+^ overload phenomenon exists in neurons during an epileptic attack. Calcium is known as the basic promoter of the excitatory toxic action of epilepsy. In the central nervous system, temporal and spatial undulation of Ca^2+^ at a certain frequency and amplitude is known as the Ca^2+^ oscillation. In primary cultured hippocampal neurons, synchronous primary Ca^2+^ oscillation is associated with dynamic changes of the membrane potential. The synergistic effect of NMDA receptors and voltage-dependent L-type Ca^2+^ channel activation allows the Ca^2+^ to move from the intracellular to the extracellular area, thereby modifying the shape of Ca^2+^ oscillation every time (14). High-level Ade is likely to affect the intracellular Ca^2+^ oscillation by regulating the Ca^2+^ channel current in neurons and decrease Ca^2+^ oscillation frequency and amplitude, so as to further play a role in inhibiting the pathological processes such as synchronized electrical activity between neurons and peripheral excitatory neurotransmitter release during epileptic attack, finally reduce the excitability of neural networks in the central nervous system, and inhibit epileptic attack. As a result, spontaneous synchronized Ca^2+^ oscillations are considered the electro-physiological basis of epileptic activity (13,15).

This study first observed spontaneous synchronized Ca^2+^ oscillations in primary cultured hippocampal neurons using confocal laser scanning microscopy. Based on this, the effect of Ade on Ca^2+^ oscillations was further investigated. Low-level Ade does not have a significant effect on Ca^2+^ oscillations in neurons but Ade at a concentration of 50 *μ*mol/l causes the spontaneous Ca^2+^ oscillation frequency and amplitude in hippocampal neurons to be decreased. Ade may influence intra-cellular Ca^2+^ oscillations by regulating the balance between the release and assimilation of the spontaneous intracellular Ca^2+^ stores in neurons. This effect shows some dependence on the Ade concentration. The high extracellular potassium environment allows the intracellular and extracellular potassium concentration gradient to be decreased, the negative value of the resting potential to be reduced, the threshold potential gap to be shortened and finally results in abnormal rise of the cell excitability. Potassium and Ca^2+^ are mutually antagonistic, so the influx of potassium is reduced and the Ca^2+^ influx is increased correspondingly, which can further trigger the electrical imbalance. Therefore, a high potassium solution was used in this study as the depolarization stimuli to activate voltage-gated Ca^2+^ channels. This was to facilitate the extracellular Ca^2+^ influx, accelerate Ca^2+^ circulation in the cytosol and Ca^2+^ stores and simulate the epileptic discharge model to observe the effect of Ade. The results showed that Ade at certain concentrations plays a role in inhibiting the high potassium-induced Ca^2+^ oscillations. When the Ade is above the physiological level, it will decrease the Ca^2+^ oscillation frequency and amplitude and is likely to further inhibit the pathological processes such as synchronized electrical activity between neurons and peripheral excitatory neurotransmitter release during epileptic attack, thereby finally reducing the excitability of neural network in the central nervous system, which is also consistent with the research findings of Brundege and Dunwiddie (16) and Phillis and Wu (17).

Ade has several receptors including A1, A2a, A2b and A3, which are all distributed throughout the central nervous system, as indicated by autoradiography. Research has shown that activated A3 receptors stabilize Ca^2+^ transport within the sarcoplasmic reticulum of myocardial cells and studies on the protective effect of the A3 receptor on the central nervous system, have also been reported (18). Ade at certain concentrations may, therefore, regulate intracellular Ca^2+^ oscillations by acting on A3 receptors, thereby eventually antagonizing the synchronized electrical activity between neurons during an epileptic attack.

As the most important member of the Ade receptor family, Ade receptor A1 with high affinity is highly expressed in the cerebral cortex, hippocampus and other sites. Pék and Lutz (19) proved through studies on the cerebral hypoxia model that through acting on A1 receptors, Ade allows the potassium conductance in the cell membrane to be increased and hyperpolarized and extracellular Ca^2+^ influx to be reduced, thereby inhibiting the excitability of neurons. Many studies have shown that Ade can inhibit the release of glutamate. Flint *et al* (20) recorded Ca^2+^ oscillations mediated by metabotropic glutamate receptors in embryonic cortical slices. Sohn *et al* (21) further pointed out that such short-term Ca^2+^ waves are mediated by metabotropic glutamate receptor 5. It was further hypothesized that Ade may open Ca^2+^ channels by acting on A1 receptors at certain concentrations. This results in hyperpolarization, inhibition of the excitatory effect of glutamate and regulation of the spatiotemporal changes of intracellular Ca^2+^ concentration. This eventually antagonizes the synchronized excitatory electrical activity between neurons during an epileptic attack. Proteomic mass spectrometry analysis in this study showed that when epilepsy is induced by pentrazole, there is no expression of glutamine synthetase in the cerebral cortex of Ade Al receptor gene knockout mice, compared with wild-type controls. Glutamine synthetase is the key enzyme in the glutamate/glutamine pathway. The Ade A1 receptor is likely to have important relevance with the activation of glutamine synthetase and acceleration of the metabolic hydrolysis of excitatory neurotransmitter glutamate in the brain, which also corroborates the results in this study to a certain extent.

To understand the action mechanism of Ade, it is necessary to further study the neuronal membrane surface receptor characteristics and intracellular and extracellular complex signaling pathways. This research provides a basis and lays preliminary research foundation for new clinical therapy and preventive approaches against epilepsy.
